# Histopathological Classification of Canine Cutaneous Round Cell Tumors Using Deep Learning: A Multi-Center Study

**DOI:** 10.3389/fvets.2021.640944

**Published:** 2021-03-26

**Authors:** Massimo Salvi, Filippo Molinari, Selina Iussich, Luisa Vera Muscatello, Luca Pazzini, Silvia Benali, Barbara Banco, Francesca Abramo, Raffaella De Maria, Luca Aresu

**Affiliations:** ^1^PoliTo^BIO^Med Lab, Biolab, Department of Electronics and Telecommunications, Politecnico di Torino, Turin, Italy; ^2^Department of Veterinary Sciences, University of Turin, Turin, Italy; ^3^Department of Veterinary Medical Sciences, University of Bologna, Bologna, Italy; ^4^MyLav-Laboratorio La Vallonea, Milan, Italy; ^5^Department of Veterinary Sciences, University of Pisa, Pisa, Italy

**Keywords:** computer-aided image analysis, cutaneous round cell tumors, deep learning, digital pathology, dog, mast cell tumors

## Abstract

Canine cutaneous round cell tumors (RCT) represent one of the routine diagnostic challenges for veterinary pathologists. Computer-aided approaches are developed to overcome these restrictions and to increase accuracy and consistency of diagnosis. These systems are also of high benefit reducing errors when a large number of cases are screened daily. In this study we describe ARCTA (Automated Round Cell Tumors Assessment), a fully automated algorithm for cutaneous RCT classification and mast cell tumors grading in canine histopathological images. ARCTA employs a deep learning strategy and was developed on 416 RCT images and 213 mast cell tumors images. In the test set, our algorithm exhibited an excellent classification performance in both RCT classification (accuracy: 91.66%) and mast cell tumors grading (accuracy: 100%). Misdiagnoses were encountered for histiocytomas in the train set and for melanomas in the test set. For mast cell tumors the reduction of a grade was observed in the train set, but not in the test set. To the best of our knowledge, the proposed model is the first fully automated algorithm in histological images specifically developed for veterinary medicine. Being very fast (average computational time 2.63 s), this algorithm paves the way for an automated and effective evaluation of canine tumors.

## Introduction

Canine round cell tumors (RCT) are commonly detected as cutaneous or subcutaneous lesions, albeit other visceral anatomical locations may be involved. The majority of these tumors are of hematopoietic origin, such as histiocytoma, lymphoma, mast cell tumor, and plasmacytoma (1). Among skin tumors appearing as discrete RCT, transmissible venereal tumor and cutaneous melanoma are included as well (2). Also, melanomas are easily mistaken with other tumor histotypes when poorly or non-pigmentated (3). Morphologically, well-differentiated RCT have unique features that help to identify the specific histotype and the histological interpretation is based on cell morphology in addition to shape and spatial distribution through the pattern analysis. Other distinguishing morphological features are the lack of cellular junctions resulting in discrete occurring cells rather than cohesive aggregates or loose aggregates of cells associated with extracellular matrix (4). In contrast, poorly differentiated RCT can be very difficult to identify by histopathology alone and phenotypic marking by immunohistochemistry or flow cytometry is often required to obtain a final diagnosis (5). The diagnostic antibody panel to differentiate canine RCT includes highly specific markers such as CD3, CD20, PAX-5, MEL-A, PNL2, IBA-1, or MUM-1 and other antibodies constitutively expressed by a wide range of cells such as MHCII, CD18 or E-cadherin (1, 6).

Computer-aided approaches in digital pathology (DP) are now recognized as valid methods in order to achieve reproducible results, improving classification accuracy and reducing variability in interpretations (7). Due to their powerful learning ability and advantages in dealing with complex patterns, deep learning algorithms have rapidly become the main methodology for analyzing medical images, especially in the field of DP (8, 9). The variety of image analysis tasks in DP includes classification (e.g., cancerous vs. healthy tissue), detection (e.g., mitosis counting), and segmentation (e.g., cell nuclei segmentation).

Inspired by the very promising performance achieved by deep learning methods in human pathology (10), we have designed a fully automated algorithm for the analysis of veterinary histopathological images. In particular, we took advantage of a specific class of deep learning methods, named Convolutional Neural Networks (CNN), to classify the most frequent canine cutaneous RCT. We first aimed to validate the suitability of the model in terms of diagnostic accuracy, speed, and confidence to classify mast cell tumors, melanomas, plasmacytomas, histiocytomas and lymphomas. In addition, we determined the ability of the CNN in predicting the Patnaik grade in mast cell tumors to prospectively consider its application in the clinic.

## Materials and Methods

### Case Selection

Surgical biopsies of the five most frequent RCT in canine species were retrospectively selected from the archive of the following veterinary laboratories: (i) MYLAV-Laboratorio La Vallonea, Milan; (ii) Department of Veterinary Medicine, University of Bologna, (iii) Department of Veterinary Sciences, University of Turin, and (iv) Department of Veterinary Sciences, University of Pisa. Tumors were available as formalin-fixed paraffin embedded tissue sections routinely stained in automation (Supplementary Table 1) with hematoxylin and eosin (HE). The selected cutaneous RCT were mast cell tumors, histiocytomas, melanomas, plasmacytomas, and T-cell lymphomas. For all the cases, the initial routine diagnosis was at least 1 year prior to the start of the study to exclude revisions by the pathologists and the diagnoses were always supported by immunohistochemistry to differentiate the cell origin. The list of antibodies employed to characterize each tumor is reported in Supplementary Table 2. Digital images were scanned with a magnification of x400 (conversion factor: 0.233 μm/pixel) using a Hamamatsu NanoZoomer S210 Digital slide scanner. Finally, European board-certified pathologists and pathologists with long expertise reviewed the diagnoses and selected up to five images with a fixed dimension of 2560x1920 from each HE slides. These images were selected as the most representative regions of the histological tumor.

### Design of the Deep Learning Model for RCT Classification

**Figure 1:**
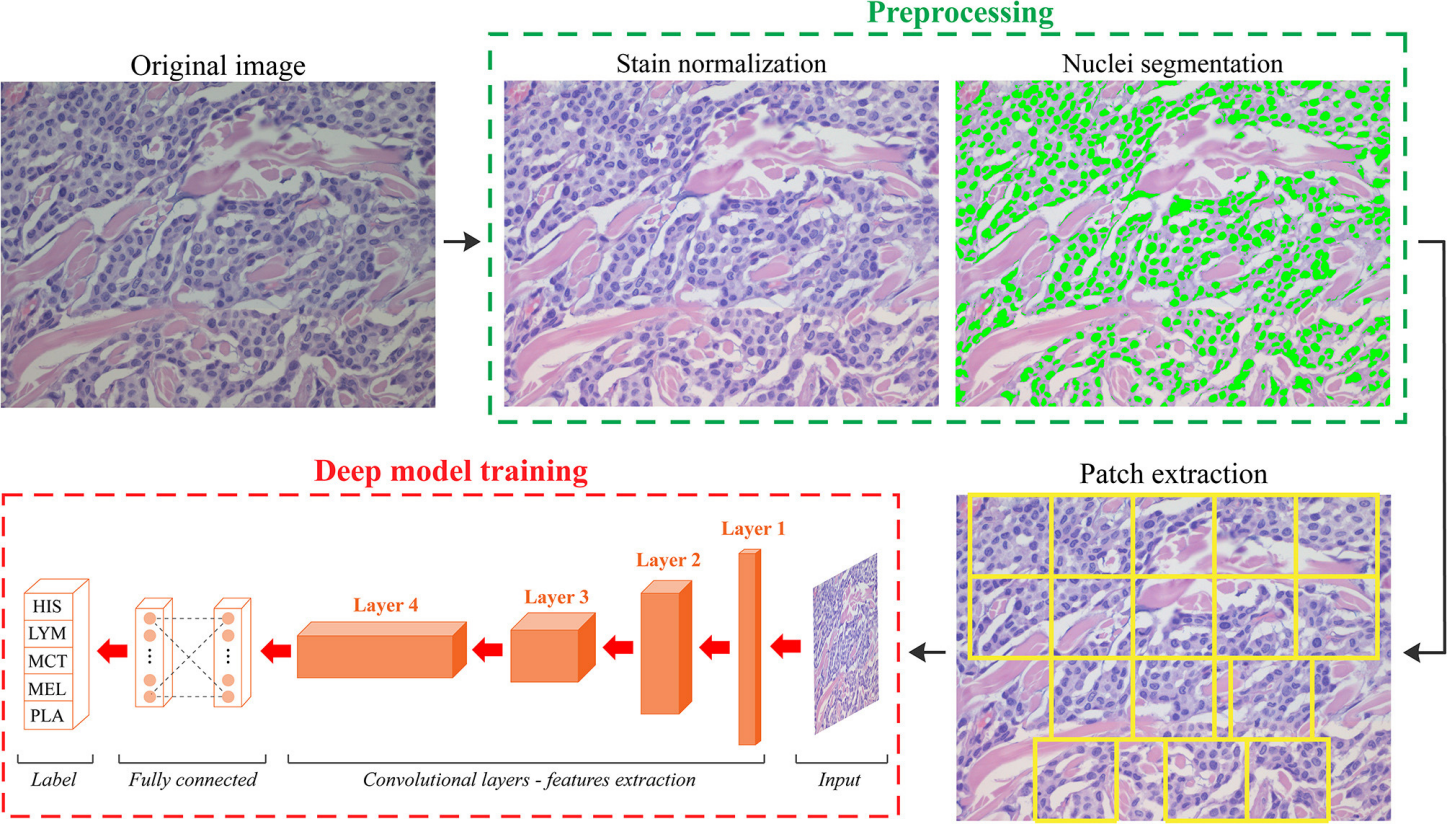
Schematic representation of the ARCTA algorithm. Starting from the original RGB image, a pre-processing stage is employed to standardize the staining intensity and to detect cell nuclei. Patches are automatically extracted based on local nuclear density. Then, a deep learning model is exploited to perform canine round cell tumors (RCT) classification. HIS, histiocytoma; LYM, lymphoma; MCT, mast cell tumor; MEL, melanoma; PLA, plasmacytoma.

We developed an automated method called ARCTA (Automated Round Cell Tumors Assessment) for canine cutaneous RCT classification that is mainly based on deep learning of HE images. An illustrative description of the model is shown in Figure 1.

The algorithm was organized in two modules: (1) preprocessing and (2) network training. In details, an initial preprocessing step was performed for color normalization of the histological images as previously described in (11). Our color normalization strategy was based on stain separation. The contribution of the individual dye (i.e., Hematoxylin and Eosin) was isolated to alter the original image according to the color distribution of the reference image. As a result, all stain-normalized images had their intensity distribution mapped to match the color distribution of the reference image. After stain normalization, an improved version of MANA algorithm (Multiscale Adaptive Nuclei Analysis) was applied for nuclear segmentation (12). Since the discriminative information were encrypted in high-resolution patches obtained from each histological image, a sliding window approach was employed to train the model extracting all the relevant ones. Specifically, a segmentation-guided patch extraction was adopted to gain all the informative tiles within the images. All the non-overlapping patches (480 × 480 pixels) that showed a minimum of 20% of their area covered with nuclei were selected by ARCTA for training. During patch extraction, we adopted a dynamic stride: if a patch was eligible for extraction, then the stride was equal to the size of the patch (480 pixels) otherwise, the stride was set to only 10% of its size (48 pixels). Thus, the patches were only extracted from regions with a high density of nuclei, most likely representative of where cancer may be present (Figure 1).

The second module of the ARCTA algorithm employed a CNN approach to perform the classification task. The Deep Learning Toolbox provided by MATLAB (MathWorks, Natick, MA, USA) was used to design and implement all the deep neural networks of this work. Since image classification is a supervised learning process where a model is trained to recognize a set of target classes using labeled example images, here we used the AlexNet (13) architecture to separate the images into the five tumor classes. Also, a transfer learning strategy (14) was applied during the network's training in order to overcome the sample size of our dataset and reduce the training time (15, 16). The dataset (Table 1) was then randomly split into a training set (392 images) and a test set (24 images). There were no significant differences between the training and the test sets concerning tumor characteristics. The AlexNet was trained with a mini-batch size of 32 and an initial learning rate of 10^−3^. Binary cross-entropy and the Adam optimizer were employed as a loss and optimization function, respectively. Finally, the maximum number of epochs was set to 30, with a validation patience of 10 epochs for early stopping of the training process. The total training time was 4 h on a dedicated workstation equipped with a 3.1 GHz octa-core CPU and 32 GB of RAM.

**Table 1:** Histological classification of 117 cutaneous canine round cell tumors.

**Task**	**Tumor (# cases)**	**# Images**
Canine RCT classification	Histiocytomas (*n* = 20)	80
	T-cell lymphomas (*n* = 20)	79
	Mast cell tumors (*n* = 20)	80
	Melanomas (*n* = 37)	98
	Plasmacytomas (*n* = 20)	79

### Design of the Deep Learning Model for Grading Cutaneous Mast Cells Tumors

**Figure 2:**
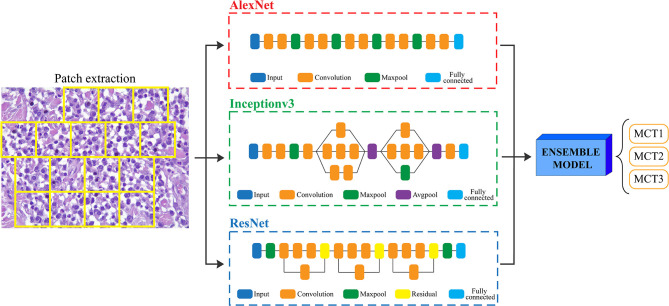
Flowchart for mast cell tumors grading. After patch extraction, three different CNNs (AlexNet, Inceptionv3, and ResNet) are employed for classification. Then, an ensemble model averages all the CNNs predictions to obtain the final grading of the image. MCT1: grade 1, MCT2: grade 2, MCT3: grade 3.

Since the prognosis for canine cutaneous mast cell tumors depends upon the tumor grade, the ARCTA algorithm was also employed to identify the Patnaik grade in mast cell tumors. Specifically, an ensemble model of three different CNN architectures was developed for this task. The first network was the AlexNet, which employs a series of convolutional layers to extract a higher-level representation of the image content. The second network was the Inceptionv3 (17) that is organized to concatenate convolutional layers having different kernel sizes. Finally, the third network architecture was the ResNet (18) that adopts skip connections and batch normalization to perform the classification. All these three networks were trained with the same hyperparameters described in the last section. During testing, each extracted patch was classified using the three CNNs and then majority voting was applied to obtain the final grading. Figure 2 illustrates the pipeline for mast cell tumors grading.

### Statistical Analysis

The overall accuracy of the deep learning model in classifying patches (patch-level accuracy) and in providing the label of the entire image (image-level accuracy) was evaluated by the classification accuracy (19). The confusion matrix and ROC curves were also calculated for the five RCT histotypes. Then, similar metrics used for RCT classification were employed to evaluate the performance for mast cell tumors grading prediction. Statistical analysis was performed using the software MATLAB equipped with the Statistics and Machine Learning Toolbox. The MATLAB implementation of the ARCTA algorithm was tested on a workstation with 3.1 GHz octa-core processor and 32-GB of RAM.

## Results

### Dataset

A total of 117 canine cutaneous RCT, including 20 histiocytomas, 20 small/medium sized T-cell lymphomas, 20 grade 2 mast cell tumors, 37 round cell-like melanomas, and 20 well-differentiated plasmacytomas, were included. Further forty-five cutaneous mast cell tumors (15 grade 1, 15 grade 2, and 15 grade 3) were included to differentiate the Patnaik grade. Tables 1, 2 summarize the dataset. The distribution of RCT and MCT cases provided from each laboratory is reported in Supplementary Tables 3, 4, respectively.

**Table 2:** Mast cell tumors grading according to Patnaik.

**Task**	**Tumor (# cases)**	**# Images**
Mast cell tumor grading	Grade 1 (*n* = 15)	76
	Grade 2 (*n* = 15)	75
	Grade 3 (*n* = 15)	80

### Canine RCT Classification

**Figure 3:**
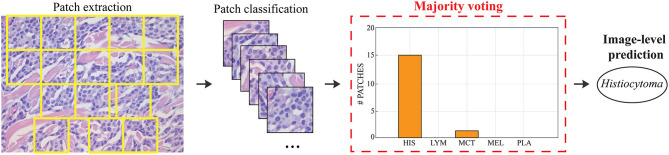
Steps for obtaining the image classification during testing. Patches are extracted based on local nuclear density. Each patch is fed into the deep learning model and the final classification is obtained using majority voting.

**Figure 4:**
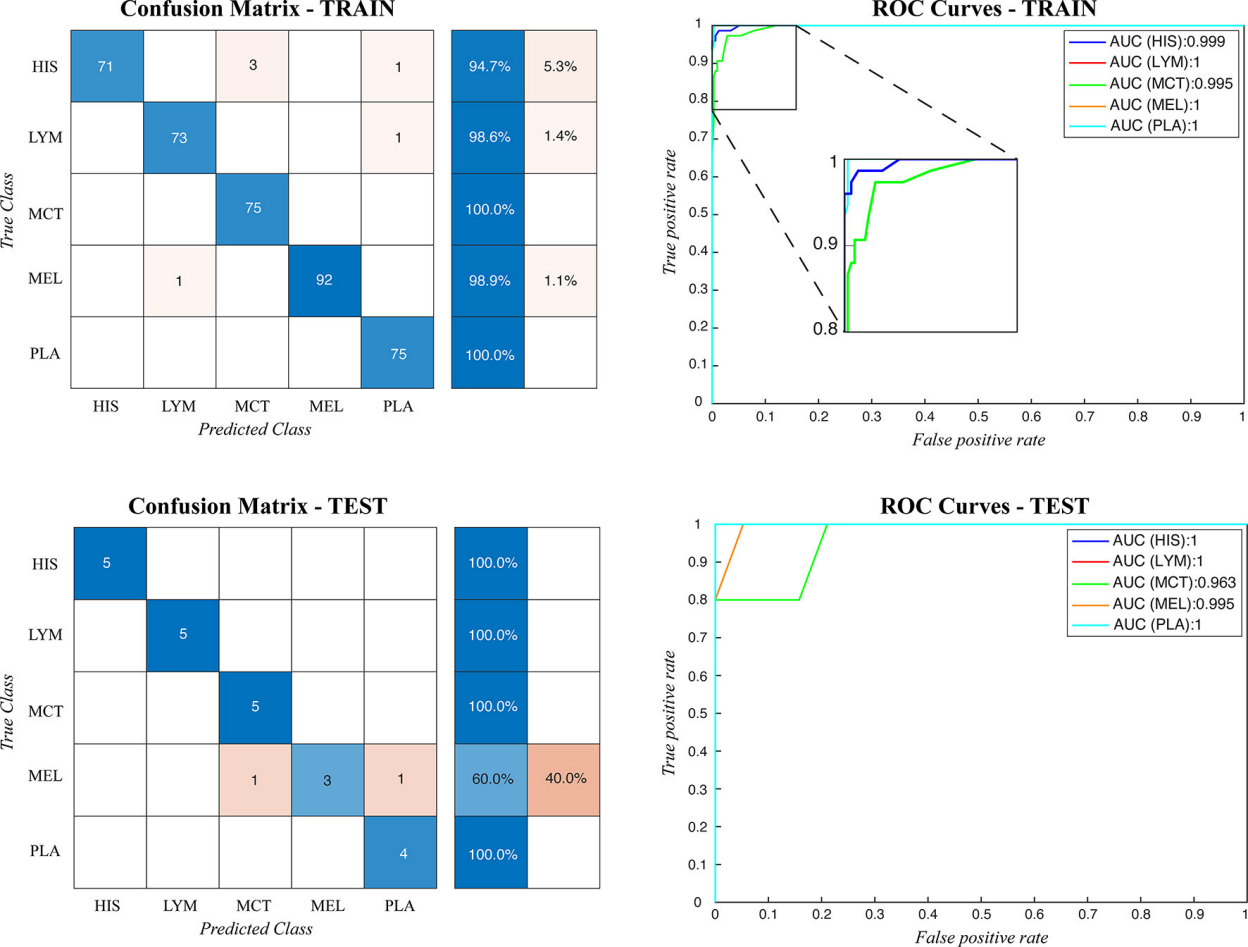
Confusion matrix and the ROC curves obtained during CRCT classification for both train and test datasets. HIS, histiocytoma; LYM, lymphoma; MCT, mast cell tumor; MEL, melanoma; PLA, plasmacytoma.

During testing, from each image the ARCTA algorithm identified cell nuclei and extracted all the relevant patches as shown in Figure 3. Then, the selected patches were fed into the AlexNet to perform the classification task. To obtain the diagnosis of the entire histological image, a voting procedure was used and the final label of the image was decided by applying a majority voting on the predicted labels obtained from the patches analysis. Figure 3 shows an example of the ARCTA algorithm during testing. The automated classification was then compared with the diagnoses assigned by the pathologists, and to evaluate the accuracy of the results a quantitative comparison was carried out. Our strategy exhibited excellent performances in classifying canine RCT with an image-level accuracy of 92.10% on train set and 87.64% on test set, respectively. In addition, the post-processing adopted to obtain the image-level classification (i.e., majority voting) allowed to further increase the performance of the deep learning model with an accuracy of 98.46% on train set and 91.66% on test set, respectively. Table 3 shows the performance of the ARCTA algorithm using train and test datasets. Figure 4 illustrates the confusion matrix and the ROC curves obtained during RCT classification. During training set evaluation, four histiocytomas were misdiagnosed as mast cell tumors (*n* = 3) and plasmacytoma (*n* = 1), one sample of each for lymphoma and melanoma was misclassified as plasmacytoma and lymphoma, respectively. During test set evaluation, melanoma samples were misdiagnosed in 40% of cases, whereas all the other tumors were classified correctly.

**Table 3:** Performance of the proposed algorithm in canine RCT classification for train and test datasets.

**Deep learning model**	**Dataset**	**Computational time (sec)**	**Patch-level accuracy**	**Image-level accuracy**
AlexNet	Train	2.63 ± 0.19	92.10%	98.46%
	Test	2.48 ± 0.22	87.64%	91.66%

### Cutaneous Mast Cell Tumor Grading

**Figure 5:**
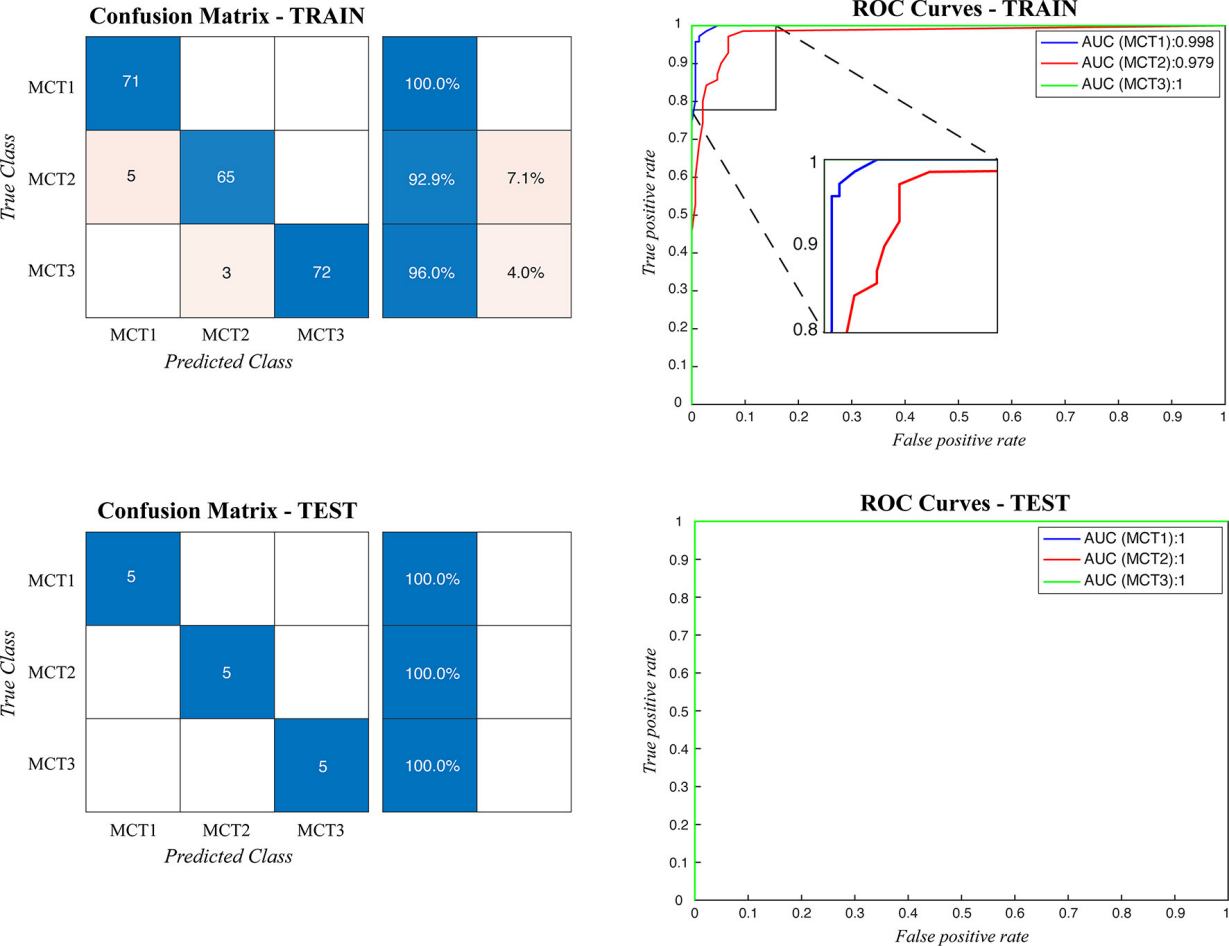
Confusion matrix and the ROC curves obtained during mast cell tumors grading for both train and test datasets. MCT1: grade 1, MCT2: grade 2, MCT3: grade 3.

When the ARCTA algorithm was employed to differentiate the mast cell tumors based on the Patnaik grade, the overall accuracy was 96.29%. The ensemble model obtained the top performance both in patch-level and image-level. Figure 5 shows the confusion matrix and the ROC curves obtained for mast cell tumors grading and Table 4 summarizes the performance of the ARCTA algorithm. Interestingly, in the train dataset, eight mast cell tumors were classified a grade lower by the model, including five mast cell tumors grade 2 and three mast cell tumors grade 3. Conversely, no misclassification was reported in test analysis.

**Table 4:** Performance of the proposed algorithm in mast cell tumors grading.

**Deep learning model**	**Dataset**	**Computational time (sec)**	**Patch-level accuracy**	**Image-level accuracy**
AlexNet	Train	2.11 ± 0.42	92.76%	93.29%
	Test	2.08 ± 0.38	93.45%	93.97%
Inceptionv3	Train	2.23 ± 0.29	91.45%	91.88%
	Test	2.27 ± 0.14	90.32%	91.53%
ResNet	Train	2.04 ± 0.27	93.11%	94.02%
	Test	2.16 ± 0.31	93.46%	94.73%
Ensemble model	Train	3.84 ± 0.22	95.32%	96.29%
	Test	3.95 ± 0.17	98.08%	100.00%

## Discussion

In several human diagnostic services, the transition of the histopathological diagnostic practice from microscope to whole slide images has paved the way for using artificial intelligence assistance systems. These innovations have increased accuracy of histological and immunohistochemical reports (20) and further resulting cost-effective (21). Only recently, veterinary diagnostic laboratories have adopted digital slides in daily routine practice opening opportunities to develop algorithms specific for canine and feline tumors (5). It's within this context that canine RCT pose a significant diagnostic challenge due to the variety of tumor histotypes with overlapping morphological and immunohistochemical features. The routinely use of flow cytometry, immunohistochemistry, and molecular biology techniques has improved the diagnosis of RCT, but several limitations are still recognized.

This study details a fully automated pipeline for the diagnosis of canine RCTs. The ARCTA algorithm was developed in dog and tested using 416 images obtained from 162 RCT, and 231 images from 45 mast cell tumors to predict Patnaik grade. Overall, for RCT classification, automatic results were similar to the original diagnosis performed by the pathologists and concordant with the immunophenotype. The algorithm showed an accuracy higher than 90% both in train and test datasets. Six misclassifications were identified in the train analysis and most of them were associated with histiocytoma diagnosis. Histologically, cutaneous histiocytomas are characterized by a high number of round cells with central or eccentrically located nuclei. Nuclei are round/oval to kidney-bean shaped, anisokaryosis can be moderate to severe, and the diagnosis without immunohistochemistry can be challenging. But more important, inflammatory cells including lymphocytes, plasma cells and macrophages can infiltrate the tumors (22). The presence of these cells may help the pathologists in performing such a diagnosis, but also alter the performance of ARCTA since population heterogeneity might influence the results of the algorithm. Similarly, a misdiagnosis with reactive histiocytosis should be considered. In our case series histiocytomas were not in regression, therefore other factors might have influenced the incorrect diagnosis by ARCTA. Conversely, the classification error of melanoma cases in test analysis was quite expected. Indeed, the variability of melanoma in both clinical presentation and histological features is well-known, being a challenge for pathologists in establishing the diagnosis and even brought it the name of “the great imitator” (23).

Since histological stain variations may adversely affect the performance and accuracy of the deep learning algorithms in DP, here we integrated a stain normalization step in the ARCTA algorithm to reduce the color variability of the histological specimens (9, 24). This practice was able to preserve the structures of the source images while forced to have a high mutual chromatic information with a template. As shown in Figure 1, the ARCTA algorithm improved the contrast between cellular structures without changing the color information of nuclei and stroma. It is important to note that the ARCTA algorithm was able to retrieve all the information used in the computational analysis from a single image of the scanned slide without analyze the entire histological section. This result suggests that the cellular features contained in a single image were sufficient for ARCTA to perform well. In addition to being accurate, the proposed method was also quite fast with an average computational time of 2.63 s for RCT classification and 3.95 s for MCT grading, respectively.

When applied to mast cell tumors the ARCTA algorithm was able to discriminate the tumor grade. The proposed ensemble model based on deep learning reached 96 and 100% accuracy in train and test, respectively. Interestingly, misclassification was always associated with a single class downgrade. Even if more data are needed to validate the algorithm for mast cell tumors, an overestimation of malignancy of the algorithm should be considered at the moment. In our experiment we decided to use the Patnaik grade to test ARCTA for three main reasons. First, this grading system has long been adopted by the veterinary pathologists compared to the recently developed 2-Tier grading system (25); second data correlating the new grading system with clinical features of dogs with mast cell tumor are still scarce and we aim here to propose a valid approach to use shortly in the clinic; third, the option to test three grades results more challenging for the algorithm, especially because grounded on nuclei only. Even if we are not able to predict the elements that have driven the performance of the algorithm, these results highlight the relevance of cellular morphology compared to other features, such as tumor depth and type of stroma, in the MCT grading using Patnaik. This is in line with the recent literature on the argument, considering more appropriate using nuclear size, chromatin pattern, nucleoli, the presence of multinucleated cells, and the number of mitotic figures to grade this tumor (25).

In conclusion, these results demonstrate that ARCTA is a robust and reliable method able to classify canine RCT with a high specificity and sensitivity and exploiting the morphological features of the neoplastic cells, but a careful revision should be considered for tumors with a more heterogenous cell population, such as histiocytoma and melanoma. Also, it's worth to note that the inclusion of more anaplastic tumor histotypes such as large cell lymphomas, poorly differentiated plasma cell tumors or high grade MCTs might diminish ARCTA performance. In future, the testing of a higher number of RCT images will help to define the features causing the misdiagnosis, but the role of the pathologists will remain fundamental since histological slides should be always evaluated first and not considered for ARCTA algorithm if other cutaneous tumors or inflammatory processes are present. To the best of our knowledge, the proposed method is the first fully automated algorithm for the diagnosis of canine tumors using HE-stained images. The advantages of ARCTA are being very fast (average computational time: 2.63 s) and to be an effective second-opinion tool for pathologists in RCT classification and mast cell tumor grading. We are currently working on an extension of this algorithm for whole-slide images processing with the aim to extract both spatial and morphological parameters and to integrate clinicopathological features, including mitotic count and immunohistochemical analysis.

## Data Availability Statement

The data supporting the conclusions of this article will be made available by the authors, under reasonable request.

## Author Contributions

MS, FM, and LA: conceptualization. MS and LA: methodology. SI and RD: formal analysis and visualization. LM, LP, SB, BB, and FA: resources and data curation. MS and LA: writing—original draft preparation. FM, SI, and RD: writing—review and editing. FM and LA: supervision. All authors have read and agreed to the published version of the manuscript.

## Conflict of Interest

The authors declare that the research was conducted in the absence of any commercial or financial relationships that could be construed as a potential conflict of interest.
