# Supplementary material for: Histopathological Classification of Canine Cutaneous Round Cell Tumors Using Deep Learning: A Multi-Center Study
Source: Front Vet Sci. 2021 Mar 26;8:640944. doi: 10.3389/fvets.2021.640944 (PMC8044886; doi:10.3389/fvets.2021.640944)
Supplement: Supplementary file 1 [file Data_Sheet_1.docx]

**Supplementary Table 1.** Automated stainer systems used in each laboratory

| **MYLAV-Laboratorio La Vallonea** | **Department of Veterinary Medicine, University of Bologna** | **Department of Veterinary Sciences, University of Turin** | **Department of Veterinary Sciences, University of Pisa** |
| --- | --- | --- | --- |
| Bioptica HPC940 | Histoline ATS200 | Leica ST5010 | Bioptica HPC940 |

**Supplementary Table 2.** Panels of antibodies used for phenotypic characterization of the round cell tumors

| **Tumor** | **Antibodies** |
| --- | --- |
| Histiocytomas | E-cadherin, MHC II, CD18 |
| T-cell Lymphomas | CD3, CD20, PAX5 and CD79 |
| Mast cell tumors | KIT |
| Melanomas | Melan A, HMB45, PNL2 |
| Plasmacytomas | MUM-1, CD3 and CD20 |

**Supplementary Table 3.** Distribution of round cell tumors provided from each institution to perform RCT classification

| **Tumor**  **(# cases)** | **MYLAV-Laboratorio La Vallonea** | **Department of Veterinary Medicine, University of Bologna** | **Department of Veterinary Sciences, University of Turin** | **Department of Veterinary Sciences, University of Pisa** |
| --- | --- | --- | --- | --- |
| Histiocytomas | 10 | 0 | 5 | 5 |
| T-cell Lymphomas | 12 | 0 | 5 | 3 |
| Mast cell tumors | 8 | 12 | 0 | 0 |
| Melanomas | 10 | 0 | 15 | 12 |
| Plasmacytomas | 7 | 3 | 6 | 4 |
| *Total* | *47* | *15* | *31* | *24* |

**Supplementary Table 4.** Distribution of mast cell tumors provided from each institution to perform MCT grading

| **MCT (# cases)** | **MYLAV-Laboratorio La Vallonea** | **Department of Veterinary Medicine, University of Bologna** | **Department of Veterinary Sciences, University of Turin** | **Department of Veterinary Sciences, University of Pisa** |
| --- | --- | --- | --- | --- |
| Grade 1 | 0 | 0 | 15 | 0 |
| Grade 2 | 0 | 0 | 15 | 0 |
| Grade 3 | 0 | 0 | 15 | 0 |
| *Total* | *0* | *0* | *45* | *0* |
